# Macronutrient-Based Dietary Patterns and the Risk of Muscle Mass Decline in Middle-Aged and Older Adults: A Prospective Community-Based Cohort Study

**DOI:** 10.3390/metabo16080568

**Published:** 2026-08-11

**Authors:** Yuji Jeong, Seok-Won Son, Se-Hong Kim, Ha-Na Kim

**Affiliations:** Department of Family Medicine, St. Vincent’s Hospital, College of Medicine, The Catholic University of Korea, Seoul 06591, Republic of Korea; yj.jeong@catholic.ac.kr (Y.J.); sonsw0826@gmail.com (S.-W.S.); iron1600@catholic.ac.kr (S.-H.K.)

**Keywords:** dietary patterns, high-carbohydrate diet, high-fat diet, high-protein diet, muscle mass, macronutrients

## Abstract

**Background/Objectives**: Loss of skeletal muscle mass is linked to functional decline and adverse health outcomes. Evidence on long-term associations between macronutrient-based dietary patterns and muscle mass decline remains limited. This study examined this association in a prospective cohort of Korean adults aged 40–69 years. **Methods**: Participants were categorized into four groups by macronutrient energy proportion: high-carbohydrate (HCHO, carbohydrate > 65% of total energy), high-fat (HF, fat > 30%), high-protein (HP, protein > 20%), and normal diets (ND). Cox models estimated associations between dietary patterns and decline in height-adjusted skeletal muscle mass, including sensitivity analyses using stricter decline thresholds (≥3%, ≥5%, and a reduction exceeding the least significant change); linear mixed-effects models evaluated longitudinal muscle mass changes. **Results**: A total of 9516 adults were included. During a median follow-up of 5.6 years (IQR 3.8–9.8), 6971 participants developed a decline in skeletal muscle mass relative to baseline. Adjusted hazards were higher for HCHO (aHR 1.12, 95% CI 1.04–1.21) and HP (aHR 1.20, 95% CI 1.01–1.42) versus ND, whereas HF showed no significant association (aHR 0.73, 95% CI 0.46–1.15). These associations were attenuated and no longer significant under stricter thresholds. Mixed-effects models showed no significant group effects or group-by-time interactions. **Conclusions**: HCHO and HP patterns were associated with a higher risk of muscle mass decline, but this was not consistent under stricter thresholds, and longitudinal trajectories did not differ across patterns. These findings should be considered preliminary. Further studies should clarify the roles of macronutrient quality, food sources, and overall dietary patterns in muscle health.

## 1. Introduction

Body composition plays an important role in physiological function, metabolic disorders, cardiovascular diseases, and mortality [1,2]. Among the components of body composition, skeletal muscle is particularly significant as it plays a crucial role in strength, physical function, performance, and metabolic regulation [3].

Loss of skeletal muscle mass begins at midlife, with an approximate decline of 1% annually. In severe cases, this reduction can result in a total muscle mass loss of approximately 50% by the age of 80 or 90 [4], which is associated with physical and functional impairment, such as a decline in strength and exercise capacity, and negative metabolic outcomes [5,6]. Muscle mass loss can be attributed to several factors, including age, sex, nutritional insufficiency, improper physical activity, pathological conditions, hormonal influences, and various metabolic disorders [7].

Previous studies have explored the relationship between dietary patterns and muscle mass. In Japanese community-dwelling adults aged ≥ 60 years, adherence to the country-specific food guide that emphasized consuming grain, vegetables, fish, meat, milk, and fruits above the recommended levels showed a positive influence on skeletal muscle [8]. Similarly, an anti-inflammatory diet, characterized by a high intake of vegetables, fruits, and whole grains, was associated with increased muscle mass and improved muscle function over a 15-year follow-up [9]. In a study using the Korea National Health and Nutrition Examination Survey 2008–2011, participants who followed a healthy dietary pattern exhibited greater muscle mass than those who adhered to a Western dietary pattern [10].

Dietary patterns and macronutrient composition have been linked to the preservation of skeletal muscle mass, strength, and function [11,12]. In a 52-week randomized controlled trial by Wycherley et al. [11], a high-protein (HP) diet (35% energy from protein) resulted in significantly smaller losses of lean mass than a high-carbohydrate (HCHO) diet (58% energy from carbohydrate). In a cross-sectional analysis using NHANES data, dietary fat intake showed an inverted U-shaped association with skeletal muscle mass in adults aged 20–59 years, with muscle mass highest at fat intake of approximately 2 g/kg body weight/day, and lower at both lower and higher intake levels [12]. However, these studies were based on a single dietary assessment and did not classify participants according to combined macronutrient distribution. Whether repeated, pattern-based dietary classification captures this risk differently remains unclear. Moreover, prospective evidence on the associations of macronutrient-based dietary patterns, such as high-protein (HP), high-carbohydrate (HCHO), and high-fat (HF) patterns, with skeletal muscle mass decline remains limited in Asian populations. The Korea Genome Epidemiology Study (KoGES) is a large community-based cohort with a large sample size, an extended follow-up period, and repeated measurements of dietary intake and body composition, making it well suited to examine the longitudinal association between dietary patterns and muscle mass loss. We hypothesized that macronutrient-based dietary patterns would be differentially associated with the risk of skeletal muscle mass decline in this population. Therefore, this study aimed to evaluate the impact of macronutrient-differential dietary patterns on the incidence of muscle mass loss among adults aged 40–69 years, using data from KoGES.

## 2. Materials and Methods

### 2.1. Study Design and Participants

This study utilized data from the KoGES, a community-based cohort study started in 2001–2002, by the Korea Centers for Disease Control and Prevention to investigate lifestyle factors, including dietary habits, and biomarkers related to chronic diseases [13]. At enrolment (2001–2002), 10,030 adults aged 40–69 years were recruited using a two-stage cluster sampling strategy, and follow-up examinations have been conducted biennially. All procedures in the original KoGES cohort were performed in accordance with the Declaration of Helsinki and approved by the Institutional Review Board of the Korea Centers for Disease Control and Prevention, with written informed consent obtained from all participants at enrolment. The present study used anonymized KoGES data, which was approved by the Institutional Review Board of the Catholic University of Korea (IRB approval: no. VC24ZISI0151, approval date: 11 July 2024).

**Figure 1:**
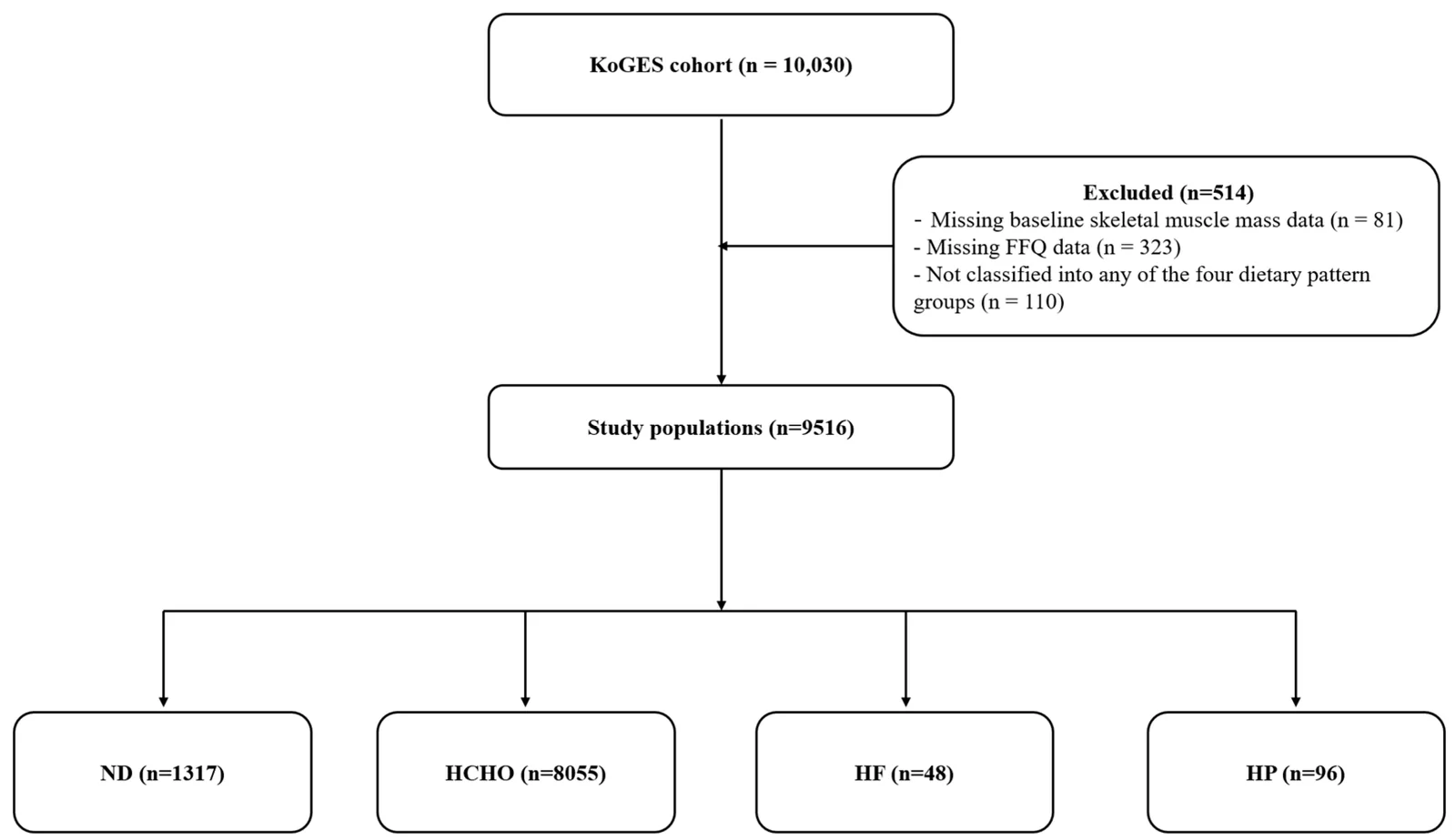
Flowchart of the study population: data from the KoGES. Abbreviations: HCHO, high-carbohydrate diet; HF, high-fat diet; HP, high-protein diet; ND, normal control diet; KoGES, Korea Genome Epidemiology Study.

Of the 10,030 KoGES baseline participants, those with missing information or values for major variables (*n* = 514) were excluded from the study. Thus, 9516 participants (4505 males and 5011 females) were included in the study and the participants were classified into four groups according to the proportions of energy from carbohydrate, protein, or fat: a high-carbohydrate (HCHO) diet, a high-fat (HF) diet, a high-protein (HP) diet, and normal control (Figure 1). The majority of participants were classified into the HCHO group, consistent with the traditionally carbohydrate-predominant composition of the Korean diet.

### 2.2. Dietary Assessments

Dietary intake was assessed by trained dietitians using a validated semi-quantitative food frequency questionnaire (FFQ) at baseline (2001–2002) and at the second follow-up examination (2005–2006). Participants reported their average frequency and portion size of each food item consumed over the previous year. Nutrient intake values were estimated using the Korean Food Composition Table provided by the Rural Development Administration in conjunction with the nutrient database of the Korea Health Industry Development Institute [14]. The FFQ used in this study was originally developed for KoGES and has been validated for the Korean population, showing good reproducibility and validity for estimating usual nutrient intakes [15].

Daily intakes of carbohydrate, protein, and fat (g/day) were calculated by summing the nutrient contributions of individual food items. The percentage of total daily energy obtained from each macronutrient was derived using standard conversion factors (4 kcal/g for carbohydrate and protein, 9 kcal/g for fat) [14]. Dietary patterns were classified according to the proportion of energy derived from each macronutrient. A high-carbohydrate (HCHO) diet was defined as >65% carbohydrate, ≤30% fat, and ≤20% protein; a high-fat (HF) diet as ≤65% carbohydrate, >30% fat, and ≤20% protein; and a high-protein (HP) diet as ≤65% carbohydrate, ≤30% fat, and >20% protein. Participants whose dietary intake met the Korean Dietary Reference Intakes (55–65% carbohydrate, 15–30% fat, and 7–20% protein) were assigned to the normal diet (ND) group [16].

### 2.3. Incidence of Muscle Mass Decline

Total skeletal muscle mass was assessed using a multi-frequency bioelectrical impedance device (InBody 3.0; Biospace Co., Seoul, Republic of Korea) available in the KoGES data [17,18]. Standardized measurement conditions regarding recent exercise, dietary intake, alcohol consumption, hydration status, and body position were applied because these factors may affect bioelectrical impedance measurements [19]. Measurements were avoided after exercise, bathing, excessive sweating, excessive fluid intake, overeating, or alcohol consumption. Before measurement, participants removed their shoes, socks, and stockings, and their palms and soles were cleaned with an electrolyte tissue. Participants were instructed to stand barefoot on the foot electrodes and hold the device handles, with their palms, thumbs, and feet positioned on eight contact electrodes. Height was measured to the nearest 0.1 cm using a calibrated digital stadiometer with participants standing upright without shoes.

The primary indicator of muscle mass was height-adjusted total skeletal muscle mass (kg/m^2^), calculated as total skeletal muscle mass (kg) divided by height squared (m^2^) [20]. Muscle mass decline was defined as any decrease in the muscle mass parameter at follow-up relative to each participant’s baseline measurement (2001–2002). Outcome ascertainment was conducted from the second to the eighth follow-up examination (2005–2006 to 2017–2018). If muscle mass decline was observed at multiple follow-up examinations, the earliest follow-up examination was used to define the event time. Participants without muscle mass decline during follow-up were censored at their last available follow-up examination.

### 2.4. Covariates

Smoking history was classified as never, former, or current. Alcohol-related data included drinking frequency, the number of drinks per occasion, types of beverages consumed, and the average daily alcohol intake (g/day of pure alcohol). Heavy drinking was defined as ≥40 g/day for men and ≥20 g/day for women. Total daily energy intake (kcal/day), estimated from the FFQ, was included as a covariate to account for differences in overall caloric consumption. Total physical activity was assessed using an interviewer-administered questionnaire covering occupational and leisure-time activities. Time spent in five activity-intensity categories was weighted using the assigned metabolic equivalent of task values: lying down, 0 MET; sedentary activity, 1.5 METs; light activity, 3 METs; moderate activity, 5 METs; and vigorous activity, 7 METs. Total physical activity was initially calculated in MET-hours per day and converted to MET-minutes per week by multiplying the value by 60 min and 7 days [21]. Household income was estimated by dividing the total monthly income by the square root of household size and was dichotomized as above or below 30% of the median. Education level was grouped into ≤6 years (elementary or less), 7–12 years (middle/high school), and ≥13 years (college or higher). Baseline comorbidities were assessed during the 2001–2002 baseline survey and defined as participants’ self-reported history of physician-diagnosed hypertension, diabetes mellitus, cardiovascular disease, or cancer. Waist circumference was measured to the nearest 0.1 cm at the midpoint between the lowest rib and the iliac crest at the end of a normal expiration without compressing the skin. Body weight was measured to the nearest 0.1 kg using a scale with participants in light clothing and without shoes. Body mass index (BMI) was computed as body weight (kg) divided by height squared (m^2^).

### 2.5. Statistical Analyses

Baseline characteristics were compared using the chi-square test or Fisher’s exact test for categorical variables, and the Kruskal–Wallis test for continuous variables. Data are reported as medians with interquartile ranges (IQR) for continuous measures and as counts with percentages for categorical measures. To evaluate the association between dietary patterns and the occurrence of muscle mass decline, extended Cox proportional hazards models with time-varying covariates were applied in both univariable and multivariable analyses. Dietary patterns were treated as time-varying exposures, and participants could contribute person-time to more than one dietary pattern category during follow-up, according to their dietary pattern in each interval. The baseline dietary pattern was used for the initial follow-up period, and the dietary pattern assessed at the second follow-up examination was used for subsequent follow-up intervals. The second follow-up dietary pattern reflected average dietary intake during the year before the muscle mass measurement at that visit. Muscle mass decline was defined as the first follow-up examination at which the muscle mass parameter was lower than the participant’s baseline measurement. Participants were followed from baseline until the first occurrence of muscle mass decline or were censored at the time of their last available follow-up examination. For incidence-rate calculations, person-years were accumulated according to the dietary pattern category observed during each follow-up interval, and events were assigned to the dietary pattern category observed during the interval in which muscle mass decline was first identified. The multivariable models were adjusted for demographic, lifestyle, and clinical factors, including age, sex, physical activity, education level, household income, and baseline muscle mass, which were treated as fixed covariates, and smoking status, alcohol use, BMI, daily energy intake, and comorbidity history, which were treated as time-varying covariates. The proportional hazards assumption was assessed in the final extended Cox model by including interaction terms between each covariate and log(time). No significant interaction between any covariate and log(time) was observed (all *p* > 0.05), indicating that there was no evidence of violation of the proportional hazard assumption. The cumulative incidence curves were estimated from the fitted extended Cox proportional hazards model with time-varying dietary patterns, and differences between dietary pattern groups were evaluated using Wald tests from the fitted model. Sensitivity analyses were conducted using alternative outcome definitions: muscle mass decline defined as a ≥3% or ≥5% reduction from baseline, and a reduction exceeding the least significant change to account for measurement error of the bioelectrical impedance device (2.32 kg in men and 1.70 kg in women; intraclass correlation coefficient 0.98 to 0.99) [17]. Linear mixed-effects models with a random intercept were used with time, group, and group-by-time interaction as fixed effects to evaluate longitudinal changes in muscle mass, adjusting for all covariates. Stratified analyses were performed according to sex, age, comorbidity, and obesity to examine whether the association between dietary patterns and muscle mass decline differed across subgroups. All statistical analyses were conducted with SAS software (version 9.4; SAS Institute, Cary, NC, USA), and statistical significance was determined at a two-sided *p* value of less than 0.05.

## 3. Results

### 3.1. Characteristics of the Study Population by Dietary Patterns at Baseline

A total of 9516 participants (4505 males and 5011 females) were included in this study with a median age of 50.0 years. The majority of participants were classified as HCHO (n = 8055). Participants with HCHO diets tended to be older, had a higher proportion of low household income, and hypertension. Participants with HP diets had a higher proportion of women and the highest levels of physical activity. Participants on the HF diet had higher proportions of current smoking, heavy drinking, the highest energy intake, and a history of myocardial infarction. Participants with a normal diet had higher proportions of college or higher education levels and the highest waist circumference and body muscle mass. During follow-up, 6971 participants (73.3%) developed muscle mass decline, and the incidence of muscle mass decline did not differ significantly across dietary patterns (*p* = 0.223) (Table 1).

### 3.2. Cumulative Impacts of Dietary Patterns on the Incidence of Muscle Mass Decline

**Figure 2:**
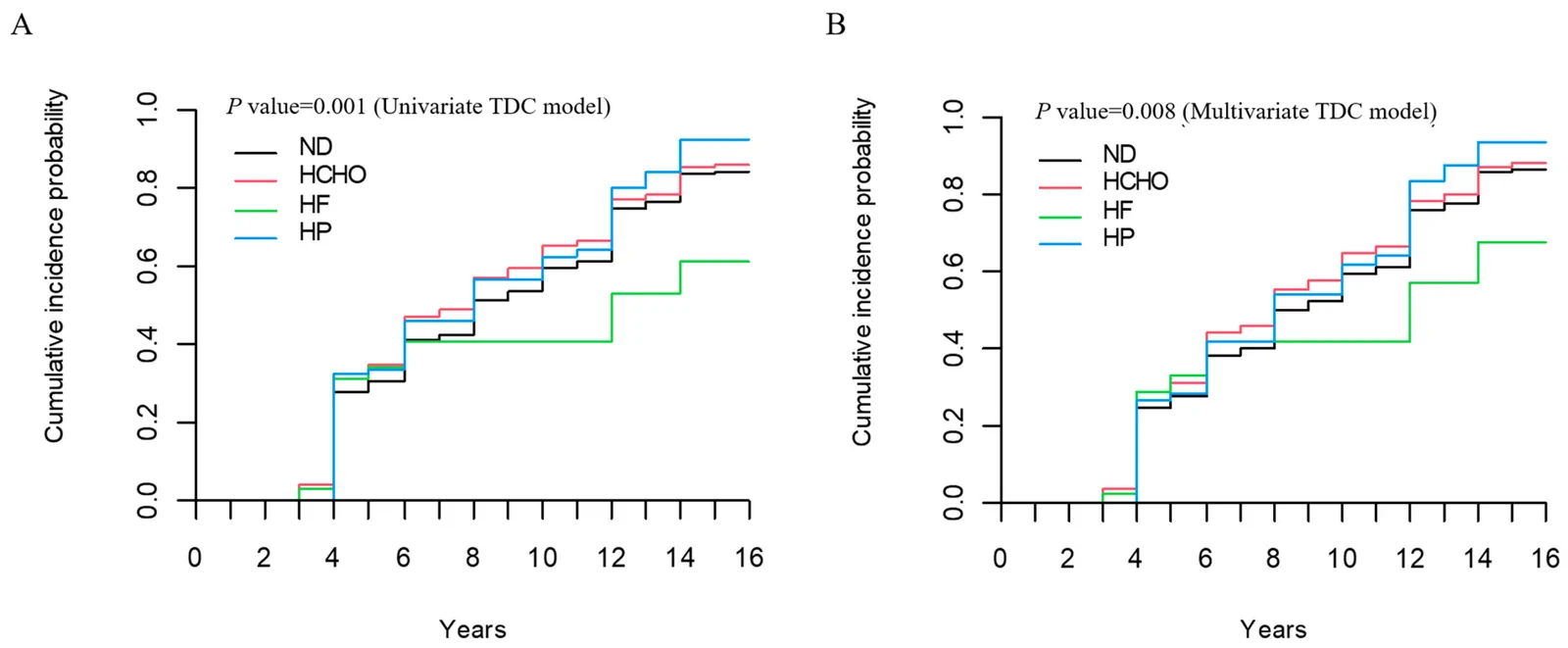
Model-estimated cumulative incidence curves derived from the extended Cox proportional hazards model with time-varying dietary patterns. (**A**) Unadjusted model. (**B**) Adjusted for age, sex, baseline muscle mass, body mass index, total energy intake, smoking status, alcohol consumption, physical activity, educational level, household income, and history of comorbidities *p* values were obtained from Wald tests of the fitted extended Cox model. Abbreviations: HCHO, high-carbohydrate diet; HF, high-fat diet; HP, high-protein diet; ND, normal control diet.

Figure 2 shows the cumulative incidence curves for reduced muscle mass based on dietary patterns using a time-dependent Cox model. In the unadjusted cumulative incidence curves, the incidence of muscle mass decline was similar across dietary patterns, although HF diet tended to show a lower cumulative incidence over time (*p* = 0.001). After adjusting for comprehensive covariates including age, sex, total energy intake, smoking status, alcohol consumption, physical activity, body mass index, educational level, history of comorbidities, household income, and baseline muscle mass, the results remained similar (*p* = 0.008).

### 3.3. Dietary Patterns as a Predictor of the Incidence of Muscle Mass Decline

Among the 9516 participants, 6971 experienced a decline in skeletal muscle mass from baseline. The median follow-up duration was 5.6 years (IQR, 3.8–9.8 years). When the outcome was defined as any decline, the incidence rates were 8.67, 11.33, 6.23, and 11.82 per 100 person-years in the ND, HCHO, HF, and HP groups, respectively. In the unadjusted extended Cox models, the HCHO and HP dietary patterns were associated with a higher risk of muscle mass decline than the ND pattern (HR, 1.12; 95% CI, 1.05–1.20 and HR, 1.21; 95% CI, 1.02–1.44, respectively). These associations remained statistically significant after adjustment for all covariates (adjusted HR [aHR], 1.12; 95% CI, 1.04–1.21 for HCHO and aHR, 1.20; 95% CI, 1.01–1.42 for HP) (Table 2).

**Figure 3:**
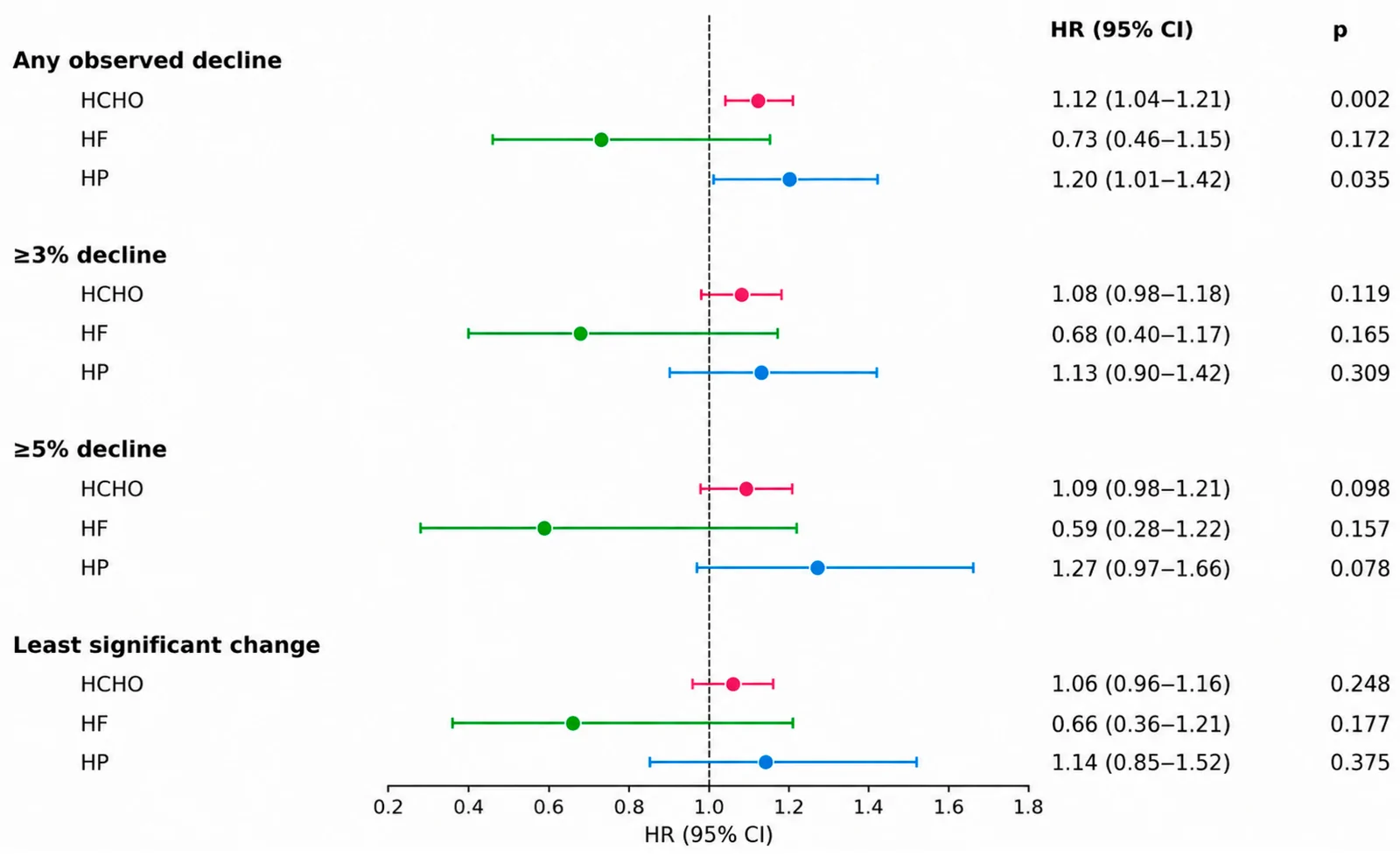
Associations between dietary patterns and skeletal muscle mass decline across outcome definitions. Any observed decline was defined as the first follow-up measurement at which height-adjusted skeletal muscle mass was lower than its baseline value. Sensitivity analyses defined the outcome as the first occurrence of muscle mass decline of ≥3%, muscle mass decline of ≥5%, or muscle mass decline exceeding the least significant change threshold (2.32 kg for male, 1.70 kg for female). Adjusted for age, sex, total energy intake, smoking status, alcohol consumption, physical activity, body mass index, educational level, history of comorbidities, household income, and baseline muscle mass. Abbreviations: CI, confidence interval; HCHO, high-carbohydrate diet; HF, high-fat diet; HP, high-protein diet; HR, hazard ratio; ND, normal diet.

The associations were attenuated when muscle mass decline was defined using more stringent thresholds. For a decline of ≥3%, neither the HCHO pattern (aHR, 1.08; 95% CI, 0.98–1.18) nor the HP pattern (aHR, 1.13; 95% CI, 0.90–1.42) was significantly associated with the outcome. Similarly, for a decline of ≥5%, both HCHO pattern (aHR, 1.09; 95% CI, 0.98–1.21) and HP pattern (aHR, 1.27; 95% CI, 0.97–1.66) did not show a statistically significant association. When muscle mass decline was defined as a reduction exceeding the least significant change, the associations were similarly attenuated for both the HCHO pattern (aHR, 1.06; 95% CI, 0.96–1.16) and the HP pattern (aHR, 1.14; 95% CI, 0.85–1.52). The HF pattern was not significantly associated with muscle mass decline under any of the outcome definitions (Figure 3 and Table S1).

**Figure 4:**
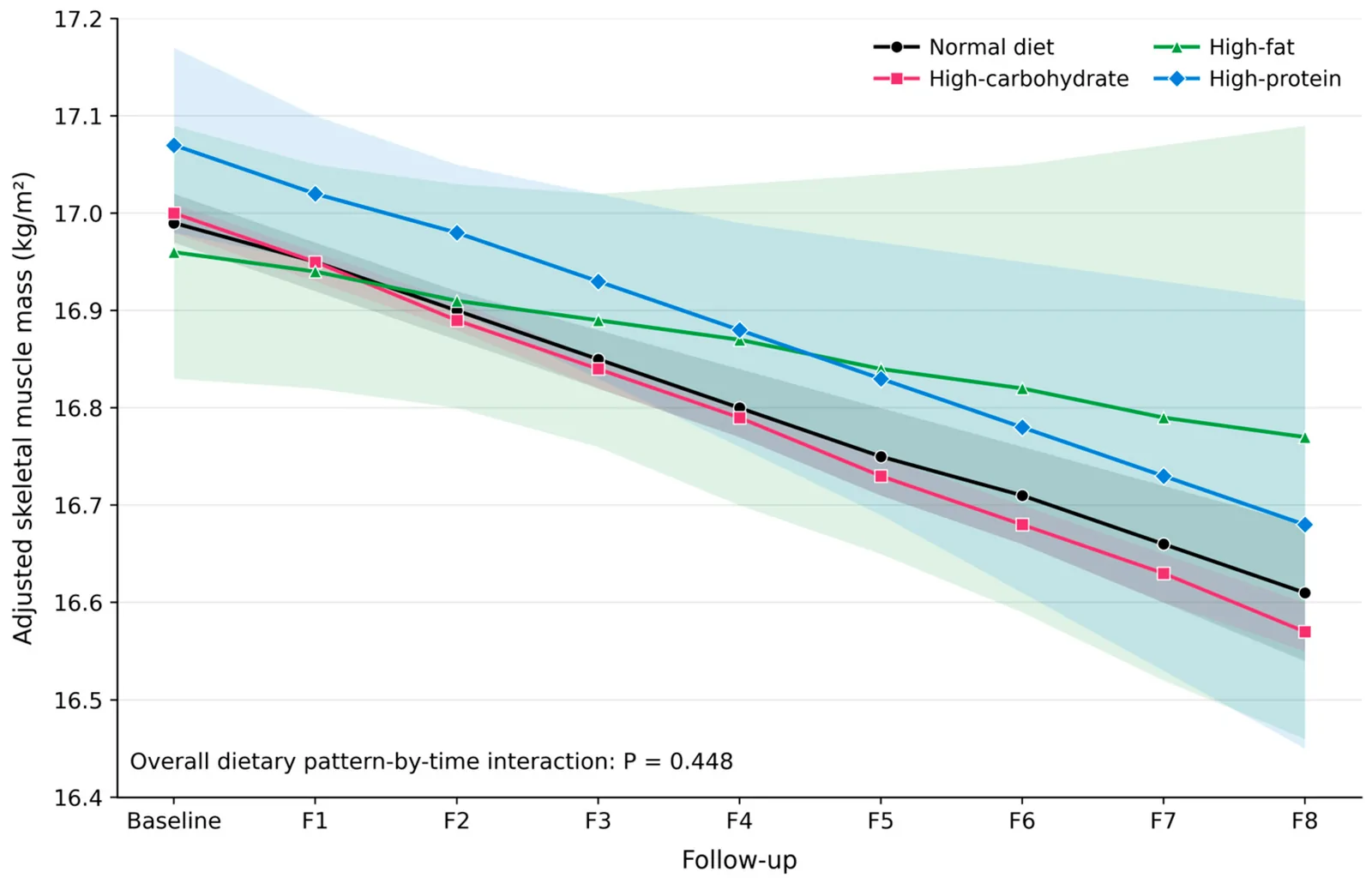
Longitudinal change in skeletal muscle mass according to dietary patterns. All estimates were obtained from the linear mixed-effects model. Baseline and follow-up values are presented as adjusted least-squares means (95% CIs), whereas group, time, and interaction effects are presented as β coefficients (95% CIs). Adjusted for age, sex, body mass index, total energy intake, smoking status, alcohol consumption, physical activity, household income, education level, and history of comorbidities. Follow-up examinations conducted biennially.

In adjusted linear mixed-effects models, height-adjusted total skeletal muscle mass decreased over time across the study population (*p* for time effect < 0.001). There was no significant overall difference in adjusted mean muscle mass among the dietary-pattern groups (*p* for group effect = 0.389). HP pattern had the highest adjusted muscle mass at baseline, while the HF pattern had the lowest; however, the HF pattern declined more gradually than the other groups and surpassed ND and HCHO by the later follow-up examinations, although this finding should be interpreted cautiously given the small size of the HF pattern. The group-by-time interaction was not significant (*p* for interaction = 0.448), indicating that the average rate of longitudinal decline in height-adjusted total skeletal muscle mass did not differ significantly among dietary-pattern groups (Figure 4).

### 3.4. Changes in Dietary Patterns as a Predictor of the Incidence of Muscle Mass Decline

Changes in dietary patterns during follow-up were associated with a risk of muscle mass decline (Table 3). Compared with the maintained ND pattern, those who changed to HCHO, HF, or HP had a higher risk of muscle mass decline (aHR 1.94; 95% CI 1.67–2.24; aHR 4.02; 95% CI 2.68–6.01; and aHR 1.65; 95% CI 1.11–2.46, respectively).

### 3.5. Subgroup Analysis for Impacts of Dietary Patterns on the Incidence of Muscle Mass Decline

In subgroup analyses stratified by sex, obesity, and comorbidity status, no significant interactions were observed (all *p* for interaction > 0.05). A significant interaction was observed for age (*p* for interaction = 0.030): among individuals aged ≥ 65 years, the HP pattern was associated with a higher risk of muscle mass decline compared with the ND pattern (aHR 2.28; 95% CI 1.04–5.01). However, this subgroup comprised a small number of participants, and the corresponding estimate should be interpreted with caution. No other significant associations were observed across the sex, obesity, or comorbidity (Table 4).

### 3.6. Non-Linear Associations Between the Proportions of Macronutrients Intake and Incidence of Muscle Mass Decline

Restricted cubic spline analyses, based on the primary outcome of any observed decline, showed no significant overall association between the percentage of energy derived from carbohydrate or protein and the risk of muscle mass decline (*p* for overall = 0.115 and 0.621) (Figure S1). There was no evidence of nonlinearity for carbohydrate or protein intake (*p* for nonlinearity = 0.213 and 0.869, respectively). In contrast, the percentage of energy derived from fat was significantly associated with the risk of muscle mass decline (*p* for overall = 0.041), with the HR decreasing progressively across the observed range, although the nonlinear association was not statistically significant (*p* for nonlinearity = 0.080).

## 4. Discussion

In this prospective community-based cohort, the HCHO and HP patterns were associated with higher hazards of any observed skeletal muscle mass decline. However, these associations were not statistically supported under the more stringent ≥3%, ≥5%, or least significant change-based definitions, and the linear mixed-effects model showed no significant differences in longitudinal skeletal muscle mass trajectories across dietary patterns. Overall, these findings suggest that the HCHO and HP patterns may be associated with the occurrence of skeletal muscle mass decline, although the observed associations appear sensitive to the magnitude and definition of decline.

The association observed for the HCHO pattern may reflect dietary and participant characteristics beyond carbohydrate energy proportion alone. Participants in this group were older and more likely to have lower household income and hypertension at baseline, and the pattern was characterized by lower absolute protein and fat intakes than the ND pattern. Previous Korean studies have also suggested that the metabolic implications of carbohydrate intake may differ according to its major food sources, particularly refined-grain consumption [22,23]. Although the models were adjusted for measured sociodemographic and clinical factors, residual confounding by dietary quality, carbohydrate sources, insulin sensitivity, inflammation, or socioeconomic characteristics cannot be excluded. Therefore, the observed association should not be interpreted as an independent adverse effect of carbohydrate intake.

The association between the HP dietary pattern and a higher hazard of any observed decline in skeletal muscle mass, particularly the exploratory finding among older adults, may appear inconsistent with the commonly proposed benefits of higher protein intake for muscle preservation. In a prospective cohort study, higher absolute intakes of total, animal, and plant protein were associated with less appendicular muscle loss in middle-aged and older adults [24]. However, protein energy proportion is not equivalent to absolute protein intake and does not capture protein sources, amino acid composition, or meal-wise distribution, all of which may be relevant to muscle health [25,26]. Moreover, because the HP group was relatively small and included particularly few older adults and events, the findings should be interpreted cautiously and require confirmation in larger studies. Thus, the observed association may reflect aspects of absolute protein intake, protein composition, meal-wise distribution, and broader dietary characteristics that were not captured by macronutrient energy proportions, rather than an adverse effect of higher protein intake itself.

Macronutrient energy proportions represent compositional characteristics of the diet, such that a higher proportion of one macronutrient necessarily corresponds to a lower proportion of one or both others [27]. Therefore, the observed associations cannot be interpreted as independent effects of carbohydrate or protein. Moreover, macronutrient proportions do not capture food sources, overall dietary quality [28,29], or other dietary and behavioral characteristics relevant to muscle maintenance [30,31]. Accordingly, the observed associations may reflect combinations of dietary and behavioral characteristics that are not captured by macronutrient energy proportions alone.

The discrepancy between the Cox and linear mixed-effects model results may reflect differences in the outcomes assessed. The Cox models evaluated the hazard of any observed decline from baseline, whereas the linear mixed-effects models assessed average longitudinal changes in muscle mass. Although the HCHO and HP patterns were associated with a higher hazard of decline, no significant differences in muscle mass trajectories were observed across dietary-pattern groups. Moreover, these associations were attenuated and no longer statistically significant when decline was defined using the ≥3%, ≥5%, or least significant change thresholds. Because the primary outcome of any decline occurred in a large majority of participants, it may have captured biological fluctuation and variability inherent in bioelectrical impedance analysis-based body-composition measurements [32]. Therefore, the main findings should be interpreted alongside the thresh-old-based sensitivity analyses.

The subgroup and dietary-pattern transition analyses should also be considered exploratory. Several estimates, including those for the HP pattern among participants aged ≥65 years and transitions involving the HF pattern, were based on few participants and events and therefore had wide confidence intervals. In addition, reverse causation cannot be excluded because declining health or muscle mass may itself have prompted dietary changes. Larger studies with more frequent dietary assessments are needed to confirm these findings and clarify the temporal direction of the associations.

The strengths of this study include its prospective, community-based design, long-term follow-up, repeated assessments of dietary intake and skeletal muscle mass, and consideration of changes in dietary patterns over time. Nevertheless, several limitations should be acknowledged. First, because appendicular muscle mass and muscle strength were unavailable in the dataset, consensus-defined sarcopenia could not be assessed [33,34]. Instead, the primary outcome was defined as any observed decline in height-adjusted total skeletal muscle mass relative to baseline. Therefore, the findings should be interpreted as associations with muscle-mass decline rather than clinically de-fined sarcopenia. The exact onset of decline could not be determined because muscle mass was assessed only at discrete follow-up examinations, and small observed declines may partly reflect variability inherent in bioelectrical impedance analysis-based measurements [32]. Second, dietary intake was assessed using FFQs administered at baseline and the second follow-up examination. Although each FFQ assessed average dietary intake during the preceding year, dietary patterns were not evaluated at every follow-up visit; consequently, intervening or subsequent dietary changes may not have been fully captured. FFQ-based dietary assessment is also susceptible to recall bias and measurement errors. Third, residual confounding cannot be excluded because information on medication use, some chronic conditions, and other factors affecting muscle metabolism was unavailable. Finally, the HF and HP groups were small, and some subgroup and dietary-pattern transition estimates were based on particularly few participants and events, limiting their precision. Therefore, these subgroup and transition findings should be considered exploratory

## 5. Conclusions

In this prospective cohort study of middle-aged and older Korean adults, HCHO and HP dietary patterns based on relative macronutrient distribution were associated with a higher risk of any observed decline in height-adjusted total skeletal muscle mass, whereas the HF pattern showed no significant association. However, these associations were attenuated and no longer statistically significant under more stringent decline thresholds, and average longitudinal muscle-mass trajectories did not differ significantly across dietary-pattern groups. These findings should therefore be considered preliminary rather than conclusive, suggesting that macronutrient proportions alone may be insufficient to explain muscle-mass preservation. Future studies should incorporate consensus-defined sarcopenia components, repeated dietary assessments, dietary quality, protein source and distribution, metabolic biomarkers, and physical activity.

## Figures and Tables

**Table 1:** Participant characteristics by baseline macronutrient-based dietary pattern.

	Total	ND	HCHO	HF	HP	*p*
*N*	9516	1317	8055	48	96	
Age (year)	50.0 (44.0–60.0)	46.0 (42.0–53.0)	52.0 (45.0–61.0)	46.5 (43.5–54.5)	50.0 (44.0–60.0)	<0.001
Sex, female (%)	52.7	43.0	54.3	37.5	57.3	<0.001
Smoking (%)						<0.001
Current	25.4	31.4	24.3	44.7	26.9	
Former	15.8	19.5	15.2	14.9	12.9	
Never	58.8	49.1	60.5	40.4	60.2	
Heavy drinking (%)	8.1	14.2	7.0	20.8	11.5	<0.001
Total physical activity (MET-min/wk)	8032.5 (4725.0–14,700.0)	7665.0 (4830.0–11,707.5)	8190.0 (4725.0–15,015.0)	8295.0 (3780.0–14,175.0)	8610.0 (5355.0–14,831.3)	0.020
Household income, low (%)	46.4	29.3	49.3	48.9	45.3	<0.001
Education (%)						<0.001
≤Elementary	33.5	15.5	36.5	14.9	35.4	
Middle to high	53.1	61.7	51.7	63.8	55.2	
≥College	13.4	22.8	11.8	21.3	9.4	
Comorbidity (%)						
Diabetes	6.7	6.6	6.6	6.3	11.5	0.314
Hypertension	15.4	11.2	16.1	12.5	14.6	<0.001
Dyslipidemia	2.4	2.4	2.4	2.1	2.1	>0.999
Stroke	1.1	0.7	1.2	0.0	2.1	0.232
Myocardial infarction	0.9	0.4	1.0	4.2	0.0	0.016
COPD	0.7	0.5	0.7	2.1	1.0	0.281
Cancer	1.0	0.6	1.1	2.1	0.0	0.226
Waist circumference (cm), Male	83.8 (78.3–88.7)	84.3 (79.7–89.7)	83.5 (78.0–88.3)	82.5 (76.7–89.2)	85.3 (80.0–90.0)	0.001
Waist circumference (cm), Female	81.0 (74.7–88.5)	78.0 (71.8–85.0)	81.7 (75.0–89.0)	85.7 (79.3–89.3)	84.0 (76.7–89.7)	<0.001
Weight (kg)	62.0 (56.0–70.0)	65.0 (58.0–72.0)	62.0 (56.0–69.0)	62.5 (57.5–69.9)	62.0 (57.0–71.5)	<0.001
BMI (kg/m^2^)	24.5 (22.5–26.5)	24.5 (22.6–26.4)	24.5 (22.4–26.5)	24.4 (21.6–25.9)	24.7 (23.0–27.7)	0.404
Energy intake (kcal/day)	1814.8 (1501.5–2207.9)	2166.8 (1757.9–2583.2)	1768.8 (1480.4–2118.0)	2459.9 (1659.7–3399.2)	2033.1 (1327.5–2741.1)	<0.001
Carbohydrate intake (g/kg/day)	5.2 (4.3–6.3)	5.0 (4.1–6.3)	5.2 (4.4–6.3)	5.1 (3.1–6.5)	4.1 (2.9–5.9)	<0.001
Fat intake (g/kg/day)	0.4 (0.3–0.6)	0.8 (0.6–1.0)	0.4 (0.3–0.6)	1.4 (1.0–2.1)	0.8 (0.5–1.2)	<0.001
Protein intake (g/kg/day)	1.0 (0.8–1.3)	1.3 (1.1–1.6)	0.9 (0.7–1.2)	1.7 (1.1–2.6)	1.6 (1.1–2.4)	<0.001
Energy from						
Carbohydrate (%)	72.6 (67.6–77.1)	62.1 (60.0–63.8)	73.9 (69.9–77.9)	50.3 (47.7–52.1)	55.4 (51.9–58.4)	<0.001
Fat (%)	14.0 (10.6–17.7)	21.7 (20.4–23.7)	13.1 (9.9–15.9)	31.9 (31.1–34.8)	23.3 (20.3–25.9)	<0.001
Protein (%)	13.4 (12.0–14.9)	16.2 (15.2–17.2)	13.0 (11.7–14.2)	17.8 (16.4–18.8)	21.3 (20.5–22.1)	<0.001
Total muscle mass (kg/m^2^)	16.7 (15.6–18.0)	17.2 (15.8–18.4)	16.7 (15.5–18.0)	16.8 (15.9–18.3)	16.7 (15.9–18.2)	<0.001
Participants with muscle mass decline (%)	73.3	72.8	73.4	60.4	75.0	0.223

Values are expressed as median (interquartile range) or percentages. Abbreviations: COPD, chronic obstructive pulmonary disease; HCHO, high-carbohydrate diet; HF, high-fat diet; HP, high-protein diet; ND, normal control diet; MET, metabolic equivalent of task; BMI, body mass index.

**Table 2:** Association between dietary patterns and the incidence of skeletal muscle mass decline.

Dietary Patterns	Event, n ^a^	Person-Years	Incidence Rate ^b^	Hazard Ratio (95% Confidence Interval)							
				Unadjusted	*p*	Model 1	*p*	Model 2	*p*	Model 3	*p*
ND	684	7885.04	8.67	1		1		1		1	
HCHO	6193	54,637.12	11.33	1.12 (1.05–1.20)	0.001	1.09 (1.02–1.17)	0.014	1.10 (1.03–1.19)	0.008	1.12 (1.04–1.21)	0.002
HF	20	321.12	6.23	0.64 (0.40–1.02)	0.059	0.62 (0.39–1.00)	0.051	0.68 (0.43–1.09)	0.109	0.73 (0.46–1.15)	0.172
HP	74	626.27	11.82	1.21 (1.02–1.44)	0.030	1.12 (0.94–1.33)	0.110	1.17 (0.98–1.40)	0.089	1.20 (1.01–1.42)	0.035

^a^ Dietary pattern was time-updated, and events were assigned to the dietary pattern observed when muscle mass decline was first identified, ^b^ Per 100 person-years. Model 1: Adjusted for age, sex, and baseline muscle mass; Model 2: Adjusted for Model 1 plus body mass index and total energy intake; Model 3: Adjusted for Model 2 plus smoking status, alcohol consumption, physical activity, educational level, household income, and history of comorbidities. Abbreviations: HCHO, high-carbohydrate diet; HF, high-fat diet; HP, high-protein diet; ND, normal control diet.

**Table 3:** Association between changes in dietary pattern and the incidence of muscle mass decline.

Dietary Pattern		Unadjusted		Model 1		Model 2		Model 3	
Baseline	Follow-Up	HR (95% CI)	*p*	HR (95% CI)	*p*	HR (95% CI)	*p*	HR (95% CI)	*p*
ND	ND	1		1		1		1	
	HCHO	1.63 (1.43–1.87)	<0.001	1.66 (1.45–1.90)	<0.001	1.92 (1.65–2.22)	<0.001	1.94 (1.67–2.24)	<0.001
	HF	4.53 (3.97–5.15)	<0.001	4.14 (2.73–6.27)	<0.001	4.05 (2.99–5.48)	<0.001	4.02 (2.68–6.01)	<0.001
	HP	1.52 (1.06–2.18)	0.022	1.49 (1.03–2.16)	0.034	1.58 (1.06–2.36)	0.024	1.65 (1.11–2.46)	0.013
HCHO	ND	1.01 (0.92–1.12)	0.789	1.04 (0.94–1.14)	0.468	1.06 (0.97–1.17)	0.218	1.05 (0.95–1.16)	0.317
	HCHO	1		1		1		1	
	HF	0.79 (0.46–1.38)	0.407	0.82 (0.48–1.41)	0.478	0.90 (0.54–1.51)	0.697	0.96 (0.59–1.58)	0.878
	HP	1.46 (1.17–1.83)	0.001	1.42 (1.17–1.71)	<0.001	1.48 (1.25–1.77)	<0.001	1.48 (1.26–1.75)	<0.001
HF	ND	5.54 (1.35–22.73)	0.018	11.14 (2.16–57.55)	0.004	7.81 (1.37–44.40)	0.020	19.00 (1.49–243.0)	0.024
	HCHO	4.74 (1.30–17.27)	0.018	8.24 (1.80–37.68)	0.007	6.73 (1.19–37.99)	0.031	12.62 (1.31–121.3)	0.028
	HF	1		1		1		1	
	HP	-	-	-	-	-	-	-	-
HP	ND	3.45 (2.15–5.53)	<0.001	4.65 (2.55–8.49)	<0.001	3.89 (2.21–6.84)	<0.001	3.20 (1.70–6.01)	<0.001
	HCHO	1.52 (0.95–2.43)	0.078	2.09 (1.27–3.43)	0.004	2.10 (1.24–3.56)	0.005	2.04 (1.21–3.45)	0.007
	HF	-	-	-	-	-	-	-	-
	HP	1		1		1		1	

Model 1: Adjusted for age, sex, and baseline muscle mass; Model 2: Adjusted for Model 1 plus body mass index and total energy intake; Model 3: Adjusted for Model 2 plus smoking status, alcohol consumption, physical activity, educational level, household income, and history of comorbidities. Each dietary pattern transition was compared with maintenance of the corresponding baseline dietary pattern. Cells with a dash indicate that hazard ratios could not be estimated because of insufficient events. Abbreviations: CI, confidence interval; HCHO, high-carbohydrate diet; HF, high-fat diet; HP, high-protein diet; HR, hazard ratio; ND, normal diet.

**Table 4:** Subgroup analyses of the association between dietary patterns and the incidence of muscle mass decline.

Variables		Dietary Patterns	Cases (n/N)	HR (95% CI)	*p*	*p* for Interaction
Sex	Male	ND	553/751	1		0.962
		HCHO	2600/3683	0.92 (0.84–1.01)	0.092	
		HF	18/30	0.97 (0.61–1.55)	0.897	
		HP	30/41	0.89 (0.62–1.30)	0.558	
	Female	ND	406/566	1		
		HCHO	3311/4372	0.99 (0.89–1.11)	0.873	
		HF	11/18	0.84 (0.45–1.56)	0.588	
		HP	42/55	1.06 (0.76–1.46)	0.740	
Age (years)	<65	ND	905/1244	1		0.030
		HCHO	5113/6912	0.97 (0.91–1.05)	0.494	
		HF	26/44	0.79 (0.53–1.18)	0.246	
		HP	65/87	0.96 (0.74–1.24)	0.752	
	≥65	ND	54/73	1		
		HCHO	798/1143	1.11 (0.83–1.48)	0.492	
		HF	3/4	2.96 (0.98–9.01)	0.055	
		HP	7/9	2.28 (1.04–5.01)	0.041	
Comorbidity	No	ND	778/1064	1		0.512
		HCHO	4515/6119	0.95 (0.87–1.02)	0.170	
		HF	25/40	0.82 (0.55–1.23)	0.339	
		HP	55/73	0.95 (0.71–1.25)	0.695	
	Yes	ND	180/252	1		
		HCHO	1394/1934	0.99 (0.84–1.17)	0.905	
		HF	4/8	1.70 (0.66–4.38)	0.273	
		HP	17/23	1.04 (0.63–1.71)	0.880	
Obesity	No	ND	540/747	1		0.290
		HCHO	3213/4568	0.93 (0.84–1.02)	0.118	
		HF	18/27	1.26 (0.79–2.00)	0.336	
		HP	38/51	0.90 (0.65–1.25)	0.518	
	Yes	ND	419/570	1		
		HCHO	2698/3487	0.98 (0.88–1.10)	0.758	
		HF	11/21	0.58 (0.31–1.07)	0.080	
		HP	34/45	1.05 (0.73–1.52)	0.783	

Cases are presented as the number of participants with decreased muscle mass/total number of participants (n/N). Adjusted for age, sex, body mass index, total energy intake, smoking status, alcohol consumption, physical activity, household income, education level, baseline muscle mass, and history of comorbidities except the variable defining each subgroup. Abbreviations: CI, confidence interval; HCHO, high-carbohydrate diet; HF, high-fat diet; HP, high-protein diet; HR, hazard ratio; ND, normal diet.

## Data Availability

Data from the Korean Genome and Epidemiology Study supporting the findings of this study are available from the Clinical & Omics Data Archive (CODA, https://coda.nih.go.kr/frt/index.do, accessed on 6 August 2026). Access to the data is restricted; however, they are available upon reasonable request and with permission from CODA.

## Supplementary Materials

The following supporting information can be downloaded at: https://www.mdpi.com/article/10.3390/metabo16080568/s1, Table S1: Association between dietary patterns and the incidence of skeletal muscle mass decline using alternative outcome definitions (sensitivity analyses). Figure S1: Restricted cubic spline curves for the associations between macronutrient energy from macronutrients and the risk of skeletal muscle mass decline.

## Abbreviations

The following abbreviations are used in this manuscript:

BMI	Body mass index
ND	Normal diet
HCHO	High carbohydrate
HF	High fat
HP	High protein
KoGES	Korea Genome Epidemiology Study
CI	Confidence interval
FFQ	Food frequency questionnaire
HR	Hazard ratio
