# Supplementary material for: Macronutrient-Based Dietary Patterns and the Risk of Muscle Mass Decline in Middle-Aged and Older Adults: A Prospective Community-Based Cohort Study
Source: Metabolites. 2026 Aug 11;16(8):568. doi: 10.3390/metabo16080568 (PMC13515610; doi:10.3390/metabo16080568)
Supplement: Supplementary file 1 [file metabolites-16-00568-s001.zip › metabolites-4448779-supplementary.pdf]

Table S1. Association between dietary patterns and the incidence of skeletal muscle mass decline using alternative outcome definitions (sensitivity analyses)

Outcomes	Dietary patterns	Event, n[a]	Person-years	Incidence rate[b]	Hazard ratio (95% confidence interval)							
					Unadjusted	p	Model 1	p	Model 2	p	Model 3	p
≥3% decline	ND	543	9745.04	5.57	1		1		1		1	
	HCHO	5134	71651.12	7.17	1.15(1.06-1.24)	0.001	1.04(0.96-1.13)	0.326	1.06(0.97-1.16)	0.212	1.08(0.98-1.18)	0.119
	HF	17	367.12	4.63	0.76(0.46-1.24)	0.268	0.73(0.44-1.21)	0.222	0.68(0.40-1.16)	0.154	0.68(0.40-1.17)	0.165
	HP	60	814.27	7.37	1.20(0.95-1.52)	0.124	1.06(0.85-1.33)	0.593	1.11(0.87-1.41)	0.396	1.13(0.90-1.42)	0.309
≥5% decline	ND	415	10867.04	3.82	1		1		1		1	
	HCHO	4156	81458.12	5.1	1.22(1.11-1.34)	<0.001	1.06(0.96-1.16)	0.238	1.08(0.97-1.19)	0.161	1.09(0.98-1.21)	0.098
	HF	13	398.12	3.27	0.83(0.48-1.43)	0.5	0.78(0.44-1.38)	0.387	0.61(0.29-1.26)	0.18	0.59(0.28-1.22)	0.157
	HP	50	906.27	5.52	1.34(1.02-1.76)	0.036	1.15(0.88-1.51)	0.302	1.25(0.95-1.64)	0.114	1.27(0.97-1.66)	0.078
Least significant change	ND	443	10605.04	4.18	1		1		1		1	
	HCHO	4293	79679.12	5.39	1.17(1.06-1.28)	0.001	1.06(0.96-1.16)	0.248	1.06(0.96-1.16)	0.259	1.06(0.96-1.16)	0.248
	HF	14	394.12	3.55	0.81(0.49-1.34)	0.406	0.78(0.45-1.34)	0.374	0.67(0.36-1.24)	0.2	0.66(0.36-1.21)	0.177
	HP	53	880.27	6.02	1.32(1.02-1.72)	0.037	1.11(0.85-1.45)	0.458	1.14(0.86-1.52)	0.366	1.14(0.85-1.52)	0.375

[a] Dietary pattern was time-updated, and events were assigned to the dietary pattern observed when muscle mass decline was first identified, [b] Per 100 person-years. Model 1: Adjusted for age, sex, and baseline muscle mass; Model 2: Adjusted for Model 1 plus body mass index and total energy intake; Model 3: Adjusted for Model 2 plus smoking status, alcohol consumption, physical activity, educational level, household income, and history of comorbidities. Abbreviations: HCHO, high-carbohydrate diet; HF, high-fat diet; HP, high-protein diet; ND, normal control diet.

Figure S1. Restricted cubic spline curves for the associations between macronutrient energy from macronutrients and the risk of skeletal muscle mass decline

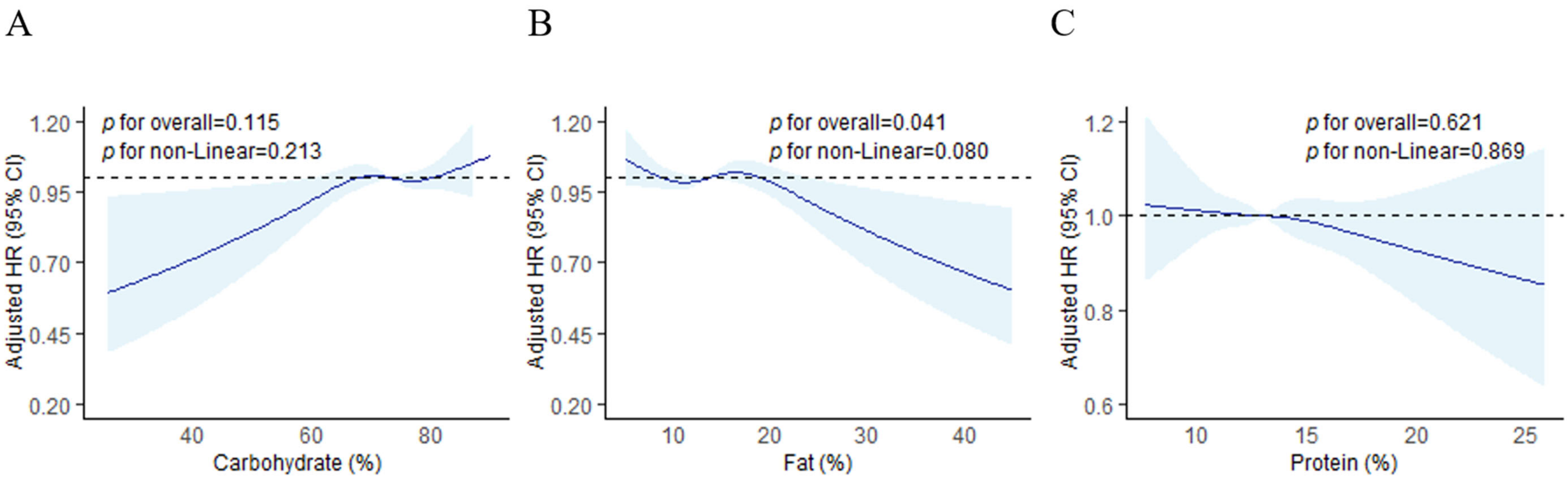

A. Energy proportion derived from carbohydrate intake. B. Energy proportion derived from fat intake. C. Energy proportion derived from protein intake. P values were determined using restricted cubic-spline regression adjusted for age, sex, baseline muscle mass, body mass index, total energy intake, smoking status, alcohol consumption, physical activity, educational level, household income, and history of comorbidities. Abbreviations: CI, confidence interval; HR, hazard ratio
